# Measuring high-efficiency perfect composite vortex beams with reflective metasurfaces in microwave band

**DOI:** 10.1515/nanoph-2024-0294

**Published:** 2024-12-02

**Authors:** Jing Hong, Mengyi Ni, Zhengping Zhang, Zheng-Da Hu, Jicheng Wang, Xiaopeng Shen, Xiong Wang, Mengmeng Li, Sergei Khakhomov

**Affiliations:** School of Science, Jiangsu Provincial Research Center of Light Industrial Optoelectronic Engineering and Technology, 66374 Jiangnan University, Wuxi 214122, China; Key Laboratory of Optoelectronic Devices and Systems of Ministry of Education and Guangdong Province, College of Physics and Optoelectronic Engineering, Shenzhen University, Shenzhen 518060, China; School of Materials Science and Physics, China University of Mining and Technology, Xuzhou 221116, China; School of Information Science and Technology, ShanghaiTech University, Shanghai 201210, China; Department of Communication Engineering, Nanjing University of Science and Technology, 210000, Nanjing, China; Departments of Optics and General Physics, Francisk Skorina Gomel State University, Sovetskaya Str. 104, Gomel 246019, Belarus

**Keywords:** perfect composite vortex beams, reflective metasurfaces, equivalent circuit model, orbital angular momentum

## Abstract

Optical vortex beams carrying orbit angular momentum have attracted significant attention recently. Perfect vortex beams, characterized by their topological charge-independent intensity profile, have important applications in enhancing communication capacity and optimizing particle manipulation. In this paper, metal-insulator-metal copper-coin type reflective metasurfaces are proposed to generate perfect composite vortex beams in X-band. We introduce the qualified equivalent circuit model based on the theory of transmission line to design the meta-atom of the structure. The experiments are performed to measure the far-field and near-field perfect composite vortex beams and evaluate their orbital angular momentum purity at different frequencies. The experimental results agree well with the theoretical predictions. This work provides new ideas and methods for generating high-quality metasurface-based perfect composite vortex beams in the microwave region, paving an ideal path for microwave communication systems, optical manipulation and radar detection.

## 1 Introduction

Optical vortex beams (OVBs) refer to a kind of special beams with helical phase wavefront exp(*ilθ*) (where *l* is the topological charge (TC), which takes arbitrary value in theory, and *θ* is the azimuth angle) [1] and ring-shaped transverse intensity distribution, which carries orbital angular momentum (OAM) [2]. Theoretically, the communication capacity can be improved by using OVBs due to orthogonality of different OAM modes [3], which raises a brand-new degree of freedom for applications in optical manipulation. Despite the precedence, the fact that the annular intensity profile of a vortex beam is highly dependent on TC increase the difficulty of information multiplexing in optical communications and limiting the flexibility of the system. Therefore, perfect vortex beams (PVBs) with the ability to maintain the ring intensity distribution constant regardless the value of TC [4] increase the data capacity in wireless communication and have been introduced as an ideal model of OVB. At present, such PVBs are mainly generated by conventional optical devices, such as axion lens [5], spatial light modulator (SLM) [4], [6], interferometer [7], and digital micro-mirror device [8], which have drawbacks of large volume, high cost, and low resolution. To break through barriers in the field of miniaturization and integration of the system, tackling these challenges typically associated with PVBs generation is urgently desirable.

Metasurfaces serve as two-dimensional metamaterials which are composed of ultrathin metallic or dielectric elements, and can locally manipulate the amplitude, phase and polarization of light in the subwavelength range, opening new perspectives for establishing micro-integrated photonic systems [9], [10], [11]. Recently, novel metasurface devices have developed rapidly, including metalenses [12], [13], holographic imaging [14], [15], vortex beam generators [16], [17], [18] and polarization converters [19], [20], [21]. The design of various functional metasurfaces paves new path for the development of wireless system [22], [23]. Generating PVBs with metasurfaces have also been reported. In 2017, Liu et al. demonstrated an approach to generating PVBs by substituting three metasurfaces for spiral phase plate, axion and Fourier lens, while the simplicity and flexibility of its design need to be improved [24]. In 2022 Liu et al. proposed a scheme of integrating three optical elements into an all-dielectric transmission metasurface to generate PVBs, thus reducing the complexity of the system [25]. In 2022, Zhang et al. generated a kind of perfect composite vortex beam (PCVB) by a single all-dielectric geometric metasurface, enhancing the functionalities of metasurfaces [26]. However, most previous works focused on generating perfect vortex beams and rarely used experiments to prove their feasibilities. Therefore, there is an urgent need for functional and feasible metasurfaces to update PVBs.

In this paper, we originally generate and experimentally demonstrate a reflective type of OAM beam generator based on copper-coin type symmetrical reflective metasurface for generating PCVBs in microwave band. The characteristics of PCVB are a rosette-like intensity pattern in the focal plane with petals that are intensively connected with TCs, while the radius remains unchanged. The extra degree of freedom is developed by introducing the concept of composite vortex beams into our metasurface design. Far-field and near-field experiments are conducted to measure the properties of metasurface-based PCVBs carrying different TCs and verify the effectiveness of the method we proposed. Measured results are in good agreement with simulation results, which shows that a single metasurface can create and engineer singularity structures. The effects of the numerical aperture (*NA*) and the focal length (*f*) for modulating the singularity structures and the purity of each OAM mode are analyzed further prove the high quality of PCVBs we produced. Our work provides a new idea and method for the modulation of the vortex field by metasurfaces in microwave band and has fabulous prospects for practical applications in wireless communication systems.

## 2 Structure and theory

**Figure 1:**
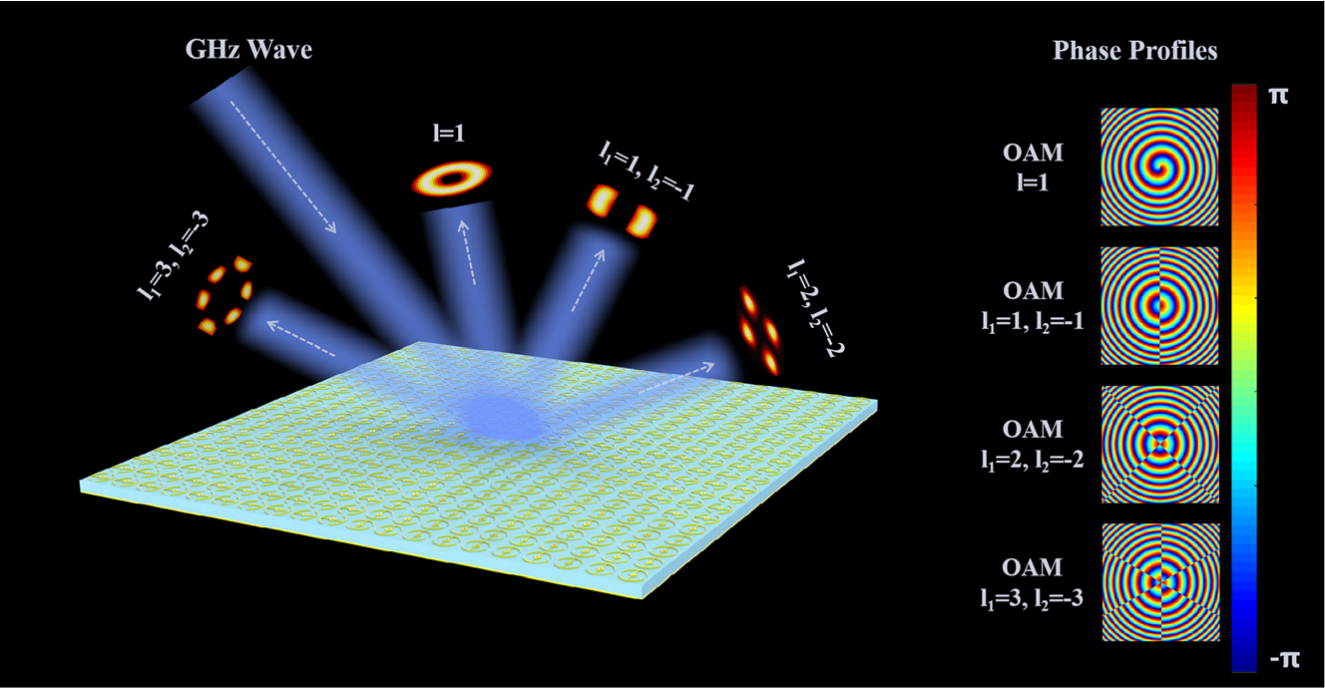
Schematic illustrations of generating PCVBs with various TCs by a MIM reflective metasurface and phase profiles of the predesigned metasurface.

Figure 1 illustrates the schematic diagram of the metasurface, which indicates that the Laguerre-Gaussian beam (LGB) incident on this metasurface can generate PVBs with different TCs superpositions, demonstrating the effect of the near-field intensity distribution of beams. The corresponding phase profiles of the PCVBs with different topological charges are shown on the right part of Figure 1. As perfect vortex beams, their intensity distribution is independent on the topological charge of the beams. Applying the Dirac delta function enables the transverse complex amplitude distribution of the beam to be expressed as follows [4]:

(1) $$E\left(\rho ,\theta \right)=\delta \left(\rho -\rho_{0}\right)\exp\left(il\theta \right),$$

where (*ρ*,*θ*) are polar coordinates in cross-section of the beam and *ρ*_0_ is the radius of the annular intensity pattern. Theoretically, PVB can be obtained by performing Fourier transform over the higher-order Bessel beams [27]. However, Bessel beams are only an ideal mathematical model in physics. Therefore, the Bessel beam is often approximated as a Bessel–Gaussian beam (BGB) in experiments and its complex amplitude can be expressed as [24]:

(2) $$E_{BG}\left(r,\varphi ,z\right)=I_{l}\left(k_{r}\rho \right)\exp\left(il\varphi \right)\exp\left(ik_{z}z\right)\exp\left(-\frac{r^{2}}{\omega_{0}^{2}}\right),$$

where *I*_1_ is the first kind of *l*th-order Bessel function, *ω*_0_ denotes the beam radius of the Gaussian beam, *k*_*r*_ and *k*_*z*_ are the radial and longitudinal wave vectors, respectively. Then, we use a lens with focal length *f* to perform Fourier transform over the BGB. In this case, Eq. (1) can be rewritten as:

(3) $$E_{pv}\left(\rho ,\theta \right)=i^{l-1}\frac{\omega_{0}}{\omega_{g}}J_{l}\left(\frac{2\rho_{r}\rho }{\omega_{g}^{2}}\right)\exp\left(il\theta \right)\exp\left(-\frac{\rho^{2}+\rho_{r}^{2}}{\omega_{g}^{2}}\right),$$

where *J*_*l*_ is the *l*th order modified Bessel function, *ω*_*g*_ and *ω*_0_ denote the waist of the BGB at *z* and *z* = 0, respectively. Since BGB can be generated from LGB by an axion lens, perfect vortex beams can also be generated by LGB passing through an axion lens and a Fourier transform lens. The transverse complex amplitude distribution of the LGB can be shown as [28]:

(4) $$\begin{array}{ll} LG & =\sqrt{\frac{2p!}{\pi \left(p+\left|l\right|\right)!}}\frac{1}{\omega \left(z\right)}{\left[\frac{\sqrt{2}p}{\omega \left(z\right)}\right]}^{\left|l\right|}\exp\left[-\frac{\rho^{2}}{\omega {\left(z\right)}^{2}}\right]L_{p}^{\left|l\right|}\\ & \;\times \left[\frac{2\rho^{2}}{\omega {\left(z\right)}^{2}}\right]\times \exp\left(il\theta \right)\exp\left[\frac{ik\rho^{2}z}{2\left(z^{2}+z_{R}^{2}\right)}\right]\\ & \;\times \exp\left[-i\left(2p+\left|l\right|+1\right)tan^{-1}\left(\frac{z}{z_{R}}\right)\right], \end{array}$$

where

(5) $$\omega \left(z\right)=\omega_{1}\sqrt{1+{\left(\frac{z}{z_{R}}\right)}^{2}},$$

where *p* is the number of radial nodes, *l* denotes the topological charge, *Lp* ^|*l*|^ is the generalized Laguerre polynomial, $Z_{R}=\pi \omega_{1}^{2}/\lambda$ is the Rayleigh range of the Gaussian envelope, *λ* is the beam wavelength and *ω*(*z*), *ω*_1_ denotes the radius of the beam at *z* and *z* = 0, respectively. Thus, PCVB can be expressed in terms of superimposing multiple LG modes with specific polarization states as:

(6) $${\left|LG\right\rangle }_{\sup}=\overset{N}{\underset{pl,\sigma }{\sum }}c{\text{e}}^{\text{i}\delta }\left|LG_{pl,\sigma }\right\rangle ,$$

where *N* denotes the total number of superposition modes, *c* and *δ* denote the amplitude coefficient and initial phase, respectively, and *σ* represents the circularly polarized state. According to Eqs. (4) with (6), we can find that PCVBs have more degrees of freedom, comparing with LGB.

To integrate and miniaturize the optical system, we can replace a helical phase plate, an axion lens, and a Fourier lens with a metasurface to generate PCVBs. The phase profiles of the three optical components can be described as [29]:

(7) $$\varphi_{\text{meta}}=\varphi_{\text{CVB}}+\varphi_{\text{axion}}+\varphi_{\text{len}},$$

(8) $$\varphi_{\text{CVB}}\left(\rho ,\theta \right)=\arg\left({\left|LG\right\rangle }_{\sup}\right),$$

(9) $$\varphi_{\text{axion}}\left(\rho ,\theta \right)=-\frac{2\pi }{\lambda }\rho \cdot NA,$$

(10) $$\varphi_{\text{len}}\left(\rho ,\theta \right)=-\frac{\pi }{\lambda f}\rho^{2},$$

where *φ*_meta_ is the phase of the meta-atom, *NA* is the numerical aperture of the axion lens, and *f* is the focal length of the Fourier lens. For a normally incident circularly polarized light, the Pancharatnam–Berry (PB) phase covering from 0 to 2*π* will be fully yielded by the rotating the meta-atom with phase *φ*_meta_.

**Figure 2:**
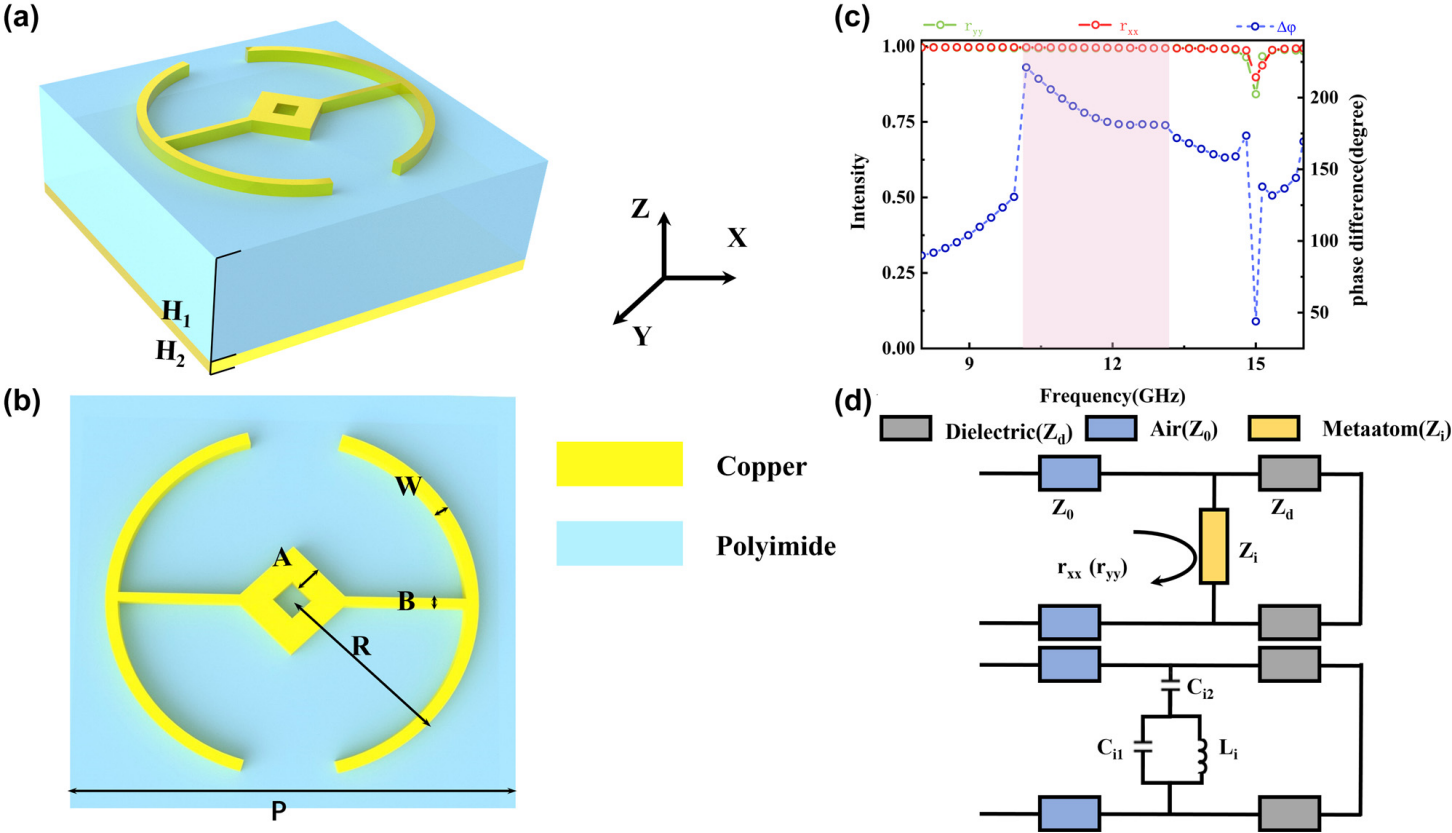
Optical properties and equivalent circuit model of the optimized meta-atom. (a) and (b) Schematic illustrations of the top view and side view of the designed unit-cell, which contains a copper meta-atom with the period of *P* = 15 mm, height of *H*_2_ = 0.2 mm a dielectric substrate with the height of *H*_1_ = 3.8 mm, and a copper ground plane. (c) Distribution of the calculated reflection amplitudes and phase difference for the *x*- and *y*-polarized light, where the pink area is the operating range of the device from 9.8 GHz to 12.9 GHz. (d) The equivalent circuit model of the single-layer metasurface unit-cell.

Figure 2 shows the structure of the copper-coin type symmetrical reflective meta-atom of the designed metasurface. It is convenient to use the form of Jones formalism to analyze the incident and scattered fields of anisotropic meta-atoms in metasurfaces [30], [31]. Usually, the reflection matrix of the reflective meta-atom which rotates an angle *θ* can be written as:

(11) $$R_{xy}\left(\theta \right)={\left[\begin{array}{cc} \cos\theta & -\sin\theta \\ \sin\theta & \cos\theta \end{array}\right]}^{-1}\left[\begin{array}{cc} r_{xx} & r_{xy}\\ r_{yx} & r_{yy} \end{array}\right]\left[\begin{array}{cc} \cos\theta & -\sin\theta \\ \sin\theta & \cos\theta \end{array}\right],$$

where $\left[\begin{array}{cc} r_{xx} & r_{xy}\\ r_{yx} & r_{yy} \end{array}\right]$ is the linear reflection coefficient of the reflective meta-atom, *r*_*xx*_ = |*r*_*xx*_|e^j*φxx*^ and *r*_*yy*_ = |*r*_*yy*_|e^j*φyy*^ correspond to the co-polarized reflection coefficients, while *r*_*xy*_ = |*r*_*xy*_|e^j*φxy*^ and *r*_*yx*_ = |*r*_*yx*_|e^j*φyx*^ correspond to the cross-polarized reflection coefficients.

Reflection coefficients with rotation angle *θ* under circularly-polarized (CP) waves incidence can be expressed as follows [32]:

(12a) $$r_{ll}=0.5\left[\left(r_{xx}-r_{yy}\right)-j\left(r_{xy}+r_{yx}\right)\right]e^{-2jk\varphi },$$

(12b) $$r_{lr}=0.5\left[\left(r_{xx}+r_{yy}\right)+j\left(r_{xy}-r_{yx}\right)\right],$$

(12c) $$r_{rl}=0.5\left[\left(r_{xx}+r_{yy}\right)-j\left(r_{xy}-r_{yx}\right)\right],$$

(12d) $$r_{rr}=0.5\left[\left(r_{xx}-r_{yy}\right)+j\left(r_{xy}+r_{yx}\right)\right]e^{2jk\varphi }.$$

From Eqs. (12a) and (12d), an abrupt phase change e^−j2*kφ*^ (e^j2*kφ*^) could be introduced by a meta-atom with a rotating angle of *kφ*. Helical phase wavefronts fronts can also be obtained from metasurface composed of rotating meta-atoms. Hence, the initial objective is to devise a meta-atom with high co-polarized reflection coefficients *r*_*ll*_ and *r*_*rr*_. When a meta-atom exhibits mirror symmetry with respect to the *yz* or *xz* plane as illustrated in Figure 2(a), the corresponding *r*_*xy*_ and *r*_*yx*_ in the Jones matrix must satisfy *r*_*xy*_ = *r*_*yx*_ = 0. To achieve high conversion from spin angular momentum (SAM) to OAM, the *r*_*xx*_ and *r*_*yy*_ of the meta-atoms should satisfy the following conditions:

(13) $$\left|r_{xx}\right|\approx \left|r_{yy}\right|\approx 1,$$

(14) $$\arg\left(r_{xx}\right)-\arg\left(r_{yy}\right)\approx \pm \pi \text{.}$$

Based on the above theory, we can design a meta-atom with period of *P* = 15 mm and other parameters are *A* = 1.4 mm, *B* = 0.5 mm, *R* = 6 mm, *H*_1_ = 3.8 mm, *H*_2_ = 0.2 mm and *W* = 0.2 mm, as shown in Figure 2(a) and (b). The proposed meta-atom system consists of a copper meta-atom array, a dielectric substrate (F4B, *ε*_*r*_ = 2.65) and a copper ground plane. The meta-atom system that we design can achieve efficient reflection from 9.8 GHz to 12.9 GHz (|*r*_*xx*_| > 0.99, |*r*_*yy*_| > 0.99) with the phase difference Δ*φ* (Δ*φ* = *φ*_*xx*_ − *φ*_*yy*_ ≈ ±*π* between the reflection amplitudes *r*_*xx*_, *r*_*yy*_), as illustrates in Figure 2(c). Figure 2(d) illustrates the corresponding equivalent circuit (EC) model, where *Z*_0_ = 377 Ω is the wave impedance of air. The wave impedance of the equivalent transmission line is *Z*_*d*_ (*Z*_0_/(*ε*_*r*_) 0.5 = 231.6 Ω). Since the meta-atom system is designed in the form of a metal ground, the equivalent circuit is modeled as a short circuit. *Z*_*i*_ (*I* ∈ {1, 2}) represents the equivalent impedance in the *x* and *y* polarized incident fields, respectively, and depends on the particular periodic metal structure printed on the dielectric. The equivalent lumped circuit models usually have been designed for the common periodic meta-atom structures, including the mesh of metallic strips (equivalent inductance) [33], the array of metallic square loops (equivalent series LC circuit) [34], [35], and metallic patch array (equivalent capacitance) [36]. Since the novelty of the meta-atom system designed in this work, it is equivalent to be a parallel circuit of the capacitance (*C*_*i*1_) and inductance (*L*_*i*_), and the effect between each other to be the capacitance (*C*_*i*2_) as shown in Figure 2(d). Based on the transmission line theory, reflection coefficients *r*_*xx*_ and *r*_*yy*_ of the corresponding circuit can be calculated by the following equation [37]:

(15) $$r_{xx}=A_{xx}e^{j\varphi_{xx}}=\frac{Z_{x}^{in}-Z_{0}}{Z_{x}^{in}+Z_{0}},$$

(16) $$r_{yy}=A_{yy}e^{j\varphi_{yy}}=\frac{Z_{y}^{in}-Z_{0}}{Z_{y}^{in}+Z_{0}},$$

(17) $$z_{i}^{in}=\frac{jZ_{d}\tan\left(\beta h\right)*\left(j\omega N_{i}+\frac{1}{j\omega C_{i1}}\right)}{jZ_{d}\tan\left(\beta h\right)+j\omega N_{i}+\frac{1}{j\omega C_{i1}}},$$

(18) $$\frac{1}{j\omega N_{i}}=\frac{1}{j\omega L_{i}}+\frac{1}{\frac{1}{j\omega C_{i2}}},$$

where $Z_{x}^{in}$ and $Z_{y}^{in}$ denote the impedance of the equivalent circuit under *x* and *y* polarized incident, *β* is the propagation constant in the F4B dielectric plate. *φ*_*xx*_ and *φ*_*yy*_ are phases of *x* and *y* polarized reflection coefficients, respectively. Calculating Eqs. (15)–(18), we can derive a set of effective capacitance and inductance values in the lumped-LC-network model (*L*_1_ = 1.696 nH, *C*_*u*1_ = 0.017 pF, *C*_*u*2_ = 0.033 pF, *L*_*v*1_ = 1.698 nH, *C*_*v*1_ = 0.017 pF, *C*_*v*2_ = 0.038 pF) for a frequency range from 10 GHz to 12 GHz.

## 3 Design and simulation results

**Figure 3:**
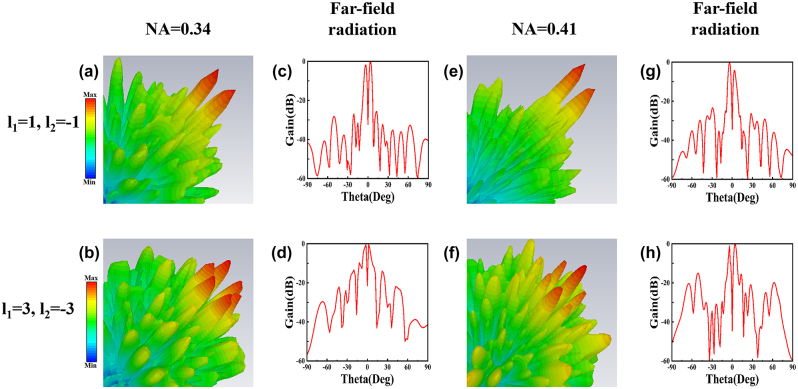
Numerically simulated 3D normalized far-field patterns and 2D normalized gains at *E*-plane of the generated PCVBs with focal length of *f* = 0.25 m and operating frequency with 10 GHz at different numerical apertures of *NA* = 0.34 and 0.41. (a) and (b) Simulated far-field patterns with TCs is *l* = ±1, *l* = ±3 at numerical apertures of *NA* = 0.34. (c) and (d) Simulated normalized gains at *E*-plane with TCs is *l* = ±1, *l* = ±3 at numerical apertures of *NA* = 0.34. (e) and (f) Simulated far-field patterns with TCs is *l* = ±1, *l* = ±3 at numerical apertures of *NA* = 0.41. (g) and (h) Simulated normalized gains at *E*-plane with TCs is *l* = ±1, *l* = ±3 at numerical apertures of *NA* = 0.41.

We utilize commercial software CST microwave studio to simulate the effectiveness of the metasurface for generating PCVBs. Figure 3 shows the numerically simulated far-field performance of the PCVBs with the TCs *l* = ±1, *l* = ±3 and *NA* changing from 0.34 to 0.41, respectively. This can be simply realized by two 300 mm × 300 mm sized metasurfaces with focal length 0.25 m and the incident frequency at 10 GHz. It can be directly observed that each intensity distribution of far-field exhibits a rosette-like appearance and petal number (*m*) are equal to *m* = |*l*_1_–*l*_2_|, as shown in Figure 3(a), (b), (e) and (f). The results of 2D gains at the focal plane are presented in Figure 3(c), (d), (g) and (h), from which we can see the linear distributions are all in the form of the hollow radiation pattern, whatever the value of TCs. Moreover, the distance between two intensity peaks becomes wider, the intensity patterns gradually become more divergent and the side lobe increases as *NA* changes from 0.34 to 0.4. This phenomenon may be affected by the relative sizes between the generated PCVBs and metasurfaces. Therefore, it is critical to strike a balance between the value of *NA* and the size of the designed metasurface for generating a high-quality PCVB with a proper size. As a result, we mainly select *NA* = 0.34 as a compromise in this work.

**Figure 4:**
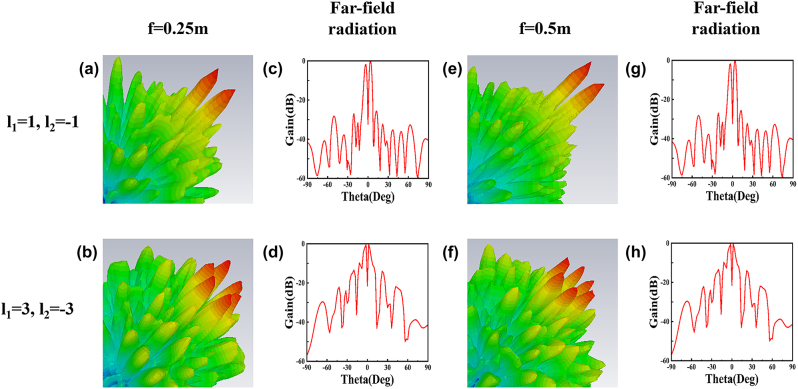
Numerically simulated 3D normalized far-field patterns and 2D normalized gains at *E*-plane of the generated PCVBs with *NA* = 0.34 at different focal lengths with *f* = 0.25 m and 0.5 m, operating at frequency with 10 GHz. (a) and (b) Simulated far-field patterns with TCs is *l* = ±1, *l* = ±3 at focal length is 0.25 m. (c) and (d) Simulated normalized gains at *E*-plane with TCs is *l* = ±1, *l* = ±3 at focal length is 0.25 m. (e) and (f) Simulated far-field patterns with TCs is *l* = ±1, *l* = ±3 at focal length is 0.5 m. (g) and (h) Simulated normalized gains at *E*-plane with TCs is *l* = ±1, *l* = ±3 at focal length is 0.5 m.

In Figure 4, we analyze the results of PCVBs generated from metasurfaces at different focal lengths of 0.25 m and 0.5 m at the central incident frequency of 10 GHz and NA is set to 0.34. These results reveal the petals of the rosette-like annular pattern and the change of the focal lengths has little influence on far-field distributions and 2D far-field radiation patterns. This phenomenon agrees well with the nature of PVBs. These results also indicate that changing TCs only influences the wavefront shape of the beam and has little effect on the radius of the vortex beams (VBs). Based on the results, the TC of the proposed PCVB can be directly read by its intensity profile instead of implementing the interference with a co-propagating Gaussian beam, simplifying topological information coding and multiplexing.

**Figure 5:**
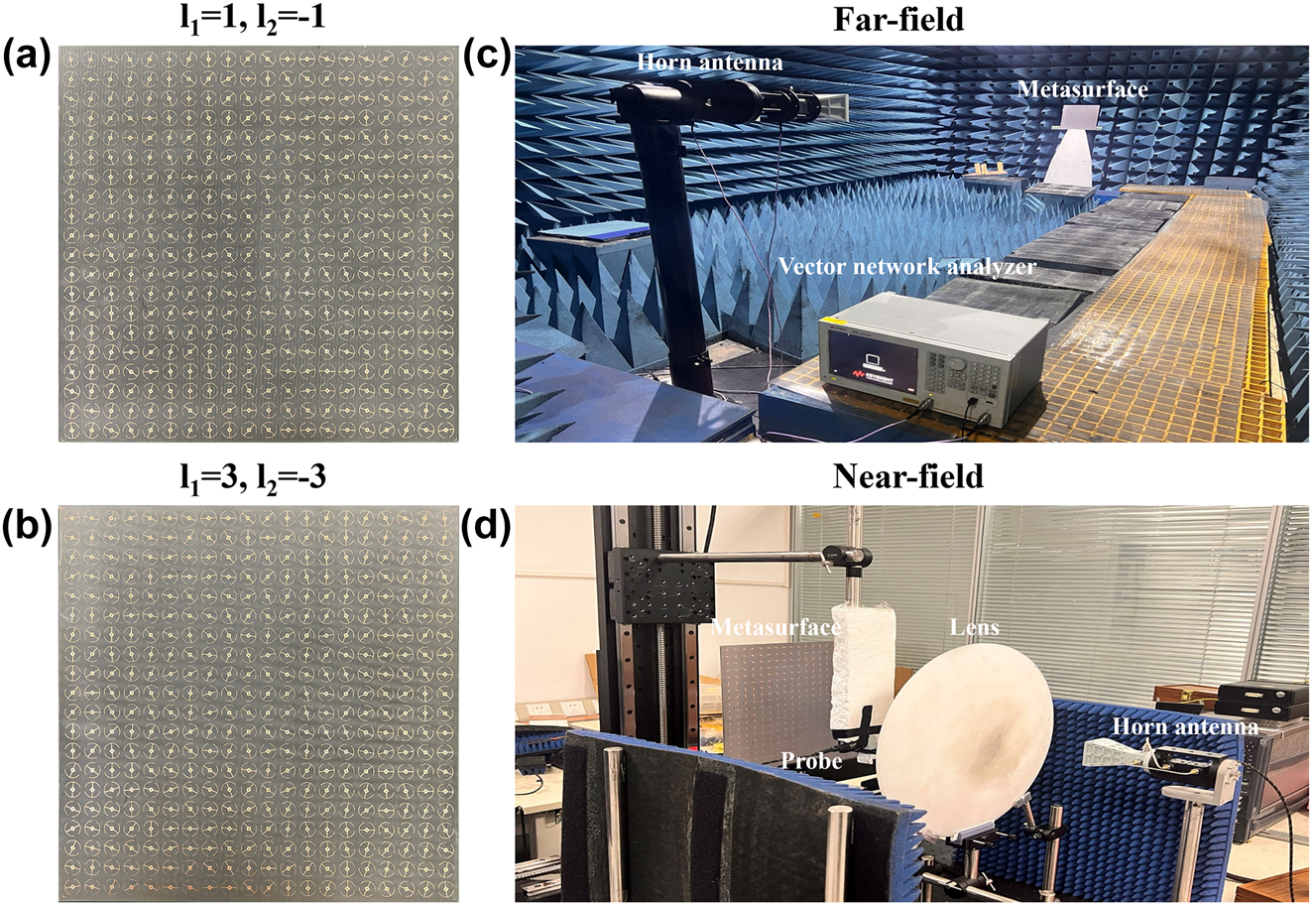
Experiment setup for measuring the far-field and near-field property of designed measurfaces. (a) and (b) Zoomed images of the fabricated measurfaces with TCs is *l* = ±1, *l* = ±3. (c) Measure environment for far-field experiments. (d) Measure environment for near-field experiments.

**Figure 6:**
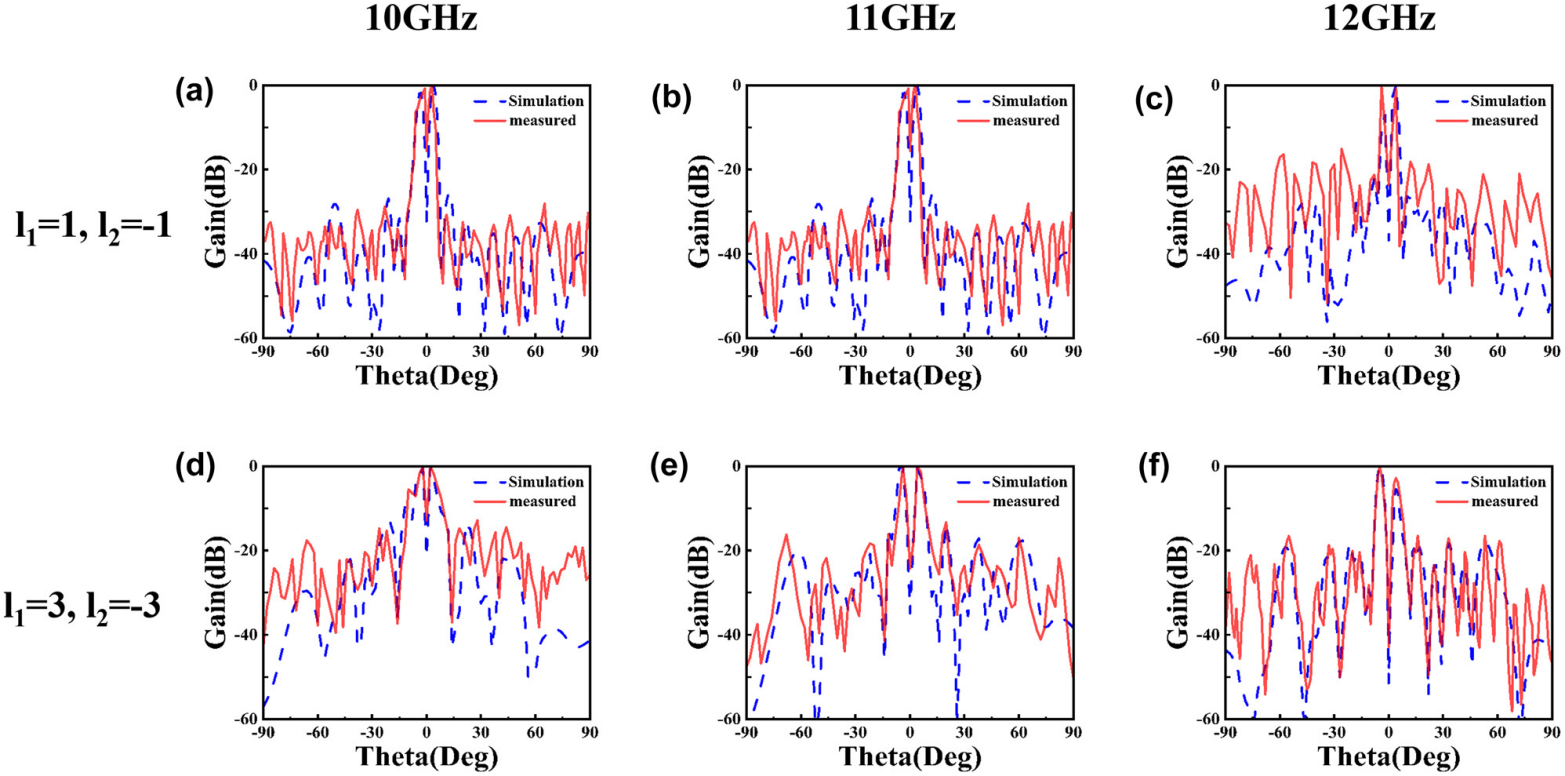
Comparisons of simulation (blue dashed line) and measured (red solid line) far-field radiation gains at different frequencies with 10 GHz, 11 GHz and 12 GHz. (a)–(c) TCs is *l* = ±1. (d)–(f) TCs is *l* = ±3.

**Figure 7:**
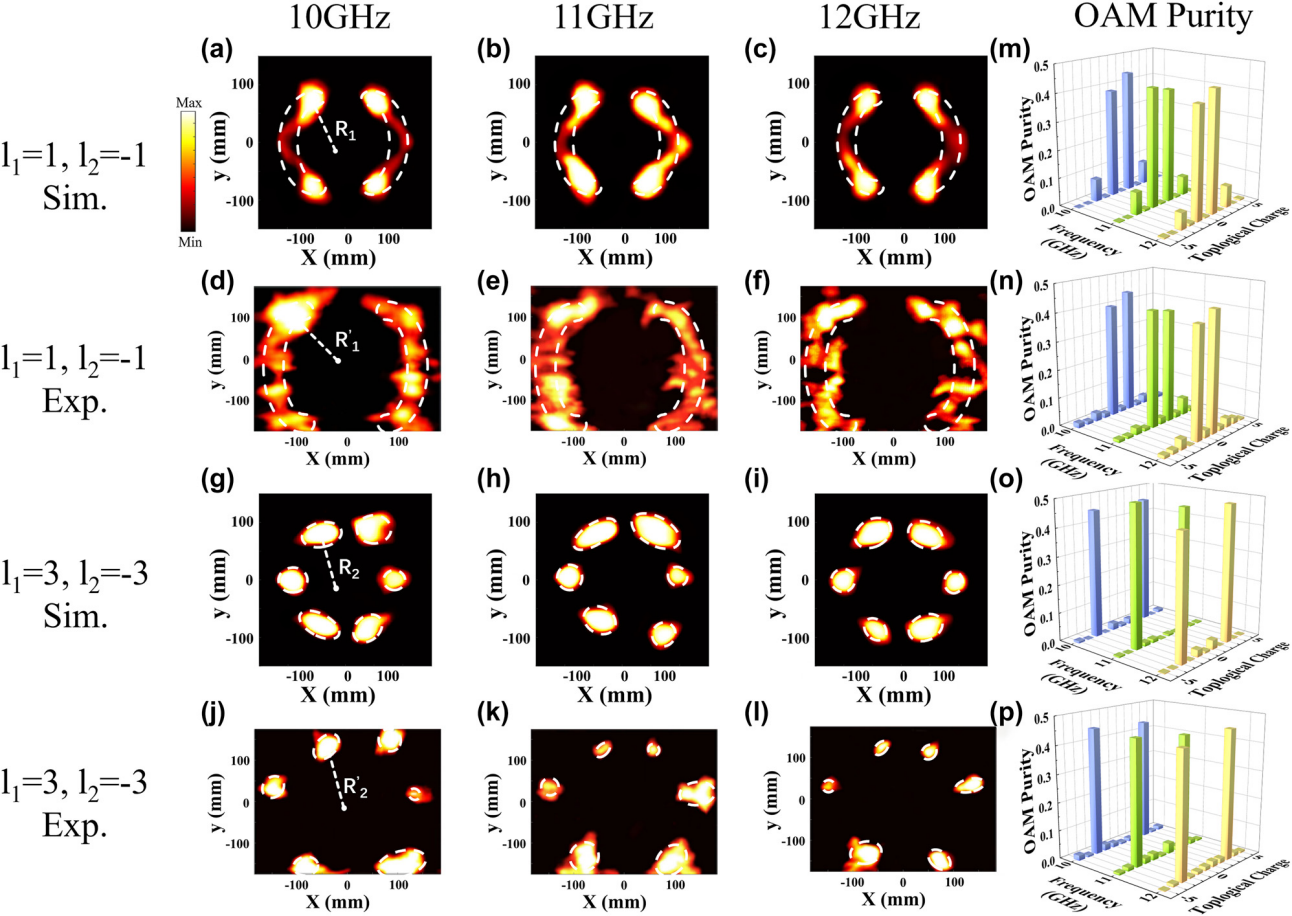
Near-field intensity distribution and purity of each OAM mode of the generated PCVBs. (a)–(f) Simulated rosette-like intensity patterns of the generated PCVBs with TCs is *l* = ±1, *l* = ±3 at different frequencies with 10 GHz, 11 GHz, and 12 GHz, using two 0.3 m × 0.3 m sized metasurfaces of *NA* = 0.34 and the focal length with *f* = 0.25 m. (g)–(l) Measured near-field intensity distribution of the generated PCVBs with TCs is *l* = ±1, *l* = ±3 at different frequencies with 10 GHz, 11 GHz and 12 GHz. (m)–(p) Calculated simulated and measured purity of each mode with TCs is *l* = ±1, *l* = ±3 at different frequencies with 10 GHz, 11 GHz, and 12 GHz.

To further validate the actual performance of our proposed metasurfaces for generating PCVBs, the two reflective metasurfaces are fabricated by printed circuit board (PCB) process and experimentally characterized employing the far-field and near-field scanning technique. The size of each fabricated metasurface is 300 mm × 300 mm, consisting of 400 meta-atoms. Figure 5 shows the photographs of the fabricated metasurfaces and the test environment of far-field and near-field measurements. We perform tests in a microwave anechoic chamber to obtain the far-field radiation characteristics of the structure and the specific experimental measurement setup is illustrated in Figure 5(c). Since the Gaussian beam can be approximated as plane wave at a distance from the light source, in the test, a LCP horn antenna is utilized as feed antenna for illuminating LCP waves on the metasurface, which is placed far enough and connected to an Agilent E5071C vector network analyzer to record far-field signal. The metasurface is placed on a turntable to provide 360° rotation in the horizontal plane. Figure 6 shows the simulated and measured far-field patterns of the proposed metasurface with different TCs *l* = ±1, *l* = ±3 at frequencies of 10 GHz, 11 GHz and 12 GHz. As illustrated, we can see the characteristic amplitude null at the center clearly, which indicates that the vortex wave is successfully generated by our designed metasurface over a broadband range. The measured results are in good agreement with the simulated results, although the measured value in the amplitude null and peaks on both sides are higher than the simulated value, which is mainly caused by imprecise fabrication and environmental disturbance. Near-field performance of the produced PCVBs also be measured by setting the near-field measurements on the optical platform to sample the spatial field distribution, as shown in Figure 5(d). The LCP horn antenna is used as the transmitting antenna and the LP waveguide antenna acts as the receiving antenna which is set at 0.25 m equaling to the designed focal length of the PCVBs. The two antennas are connected to the two ports of the PNA network analyzer (Keysight N5227A) [38]. *X*–*Y* plane can be covered by varying the position of the probe via the motion controller. The observation plane size is set as 400 mm × 350 mm and the small scanning step select 2 mm to measure the amplitude distributions at the scanning plane. The near-field experiments are favourable to obverse the rosette-like intensity distribution of PCVBs at the focal plane. Figure 7 shows the corresponding simulated and measured near-field amplitude distributions at frequencies is 10 GHz, 11 GHz, 12 GHz with TCs is *l* = ±1, *l* = ±3. Due to the number of metasurface arrays is not enough, the near-field intensity of the simulation results distribute unevenly. The performance of designed VBs could be improved by increasing the size of the metasurface. However, the intensity distributions exhibit a typical rosette-like appearance in the focal plane. The measured results also prove that the designed metasurfaces can produce PCVBs with different TCs in microwave band and agree well with the simulation results, which can verify the feasibility of our methodology. As illustrated, the simulated results of radii with *l* = ±1 are *R*_1_ = 93.1 mm, 91.4 mm, 90.1 mm at frequencies of 10 GHz, 11 GHz and 12 GHz, respectively and radii with *l* = ±3 are *R*_2_ = 93.3 mm, 91.4 mm, 90.2 mm at frequencies of 10 GHz, 11 GHz and 12 GHz, respectively. The radii of beams are approximately the same, indirectly reflecting the perfect properties of PCVBs described above and proving the PCVBs we produced have better resilience to environmental disturbances. Meanwhile, the measured results of radii with *l* = ±1 are $R_{1}^{\prime }$ = 117.9 mm, 117.4 mm, 116.8 mm at frequencies of 10 GHz, 11 GHz and 12 GHz, respectively, and radii with *l* = ±3 are $R_{2}^{\prime }$ = 118.4 mm, 118.0 mm and 116.4 mm at frequencies of 10 GHz, 11 GHz and 12 GHz, respectively. The deviations between experimental and simulated results are mainly due to imprecise fabrication, environmental disturbances, alignment errors and other experimental equipment. Our proposed PCVBs can be directly distinguished through observation, which improve the signal clarity in wireless communication, owing to their easy-to-directly-detect feature from the TC-dependent intensity petals, compared with those prior complicated approaches for optical vortex distinction [39], [40]. The petals are varying as two times of the TCs, with the increase of the TCs, while the radii of the annular intensity patterns are nearly remaining “perfect”.

The quality of the generated PCVBs is further analyzed by calculating the purity of each OAM mode recorded in the focal plane by decomposing the output electromagnetic field. The field can be expanded and represented by a linear combination consisting of basic modes [41]:

(19) $$U\left(\theta \right)=\sum C_{pl}e^{il\theta },$$

(20) $$C_{pl}=\int \underset{R}{\int }U\left(r\right)e^{-il\theta }d^{2}r,$$

where $r=\sqrt{x^{2}+y^{2}}$ , $|C_{pl}{|}^{2}$ is used to represent the purity of each mode with topological charge *l*. Histograms of results are shown in Figure 7(m)–(p). Based on the comparison of the measured and simulated results, we can clearly see that the mode weights with topological charges *l* = −1 and *l* = 1 (*l* = −3 and *l* = 3) are significantly higher than the others. Since TCs of our designed VBs are superimposed, we calculate the purity of each mode by superimposing the mode weights with topological charges *l* = −1 and *l* = 1 (*l* = −3 and *l* = 3) to assess the quality of the PCVBs. As illustrated, the simulated results of mode purity with *l* = ±1 is 82.28 %, 82.79 % and 83.51 % (*l* = ±3 is 90.71 %, 95.40 % and 92.52 %) at frequencies of 10 GHz, 11 GHz and 12 GHz, respectively. Meanwhile, the measured results of mode purity with *l* = ±1 is 83.90 %, 81.14 % and 83.02 % (*l* = ±3 is 88.58 %, 85.84 % and 89.89 %) at the above three frequencies, respectively. Simulated and measured results indicate that the OAM modes which we need occupy dominant position and the intensities of the normalized mode weights extracted to the topological charge of *l* = −1 and *l* = 1 (*l* = −3 and *l* = 3), respectively, in the focal plane are almost equal. As a result, our designed metasurfaces can generate desired PCVBs effectively and efficiently in microwave region.

## 4 Conclusions

In summary, we have proposed a MIM reflective metasurface operating in the microwave band to generate PCVBs whose intensity distribution exhibits a rosette-like appearance composed of several TC-related petals. We utilize commercial software CST microwave studio to analyze both the far-field and the near-field performance of PCVBs under different conditions validating the feasibility of this method. Based on the experiments, our design is demonstrated to perform a great functionality of generating the PCVBs at different frequencies, *NA* and focal lengths. The experimental mode purity of the PCVBs turns out to be consistent with the simulation results, which confirms the quality of the generated PCVBs. Our work provides a compact platform to generate PCVBs without a complicated optical system, which can further facilitate integrated photonics. Superposition of multiple OAM beams makes this technology very attractive for potential applications ranging from electromagnetic wave beam control to high-capacity optical communication systems.
